# Supplementary material for: Effects of Vaping Prevention Messages on Electronic Vapor Product Beliefs, Perceived Harms, and Behavioral Intentions among Young Adults: A Randomized Controlled Trial
Source: Int J Environ Res Public Health. 2022 Oct 30;19(21):14182. doi: 10.3390/ijerph192114182 (PMC9655851; doi:10.3390/ijerph192114182)
Supplement: Supplementary file 1 [file ijerph-19-14182-s001.zip › ijerph-1911785-supplementary.pdf]

Figure S1. Vape education messages (Intervention Condition), PACE Vape Messaging Study, 2020

	Alone	+Social	+Flavor
HARM	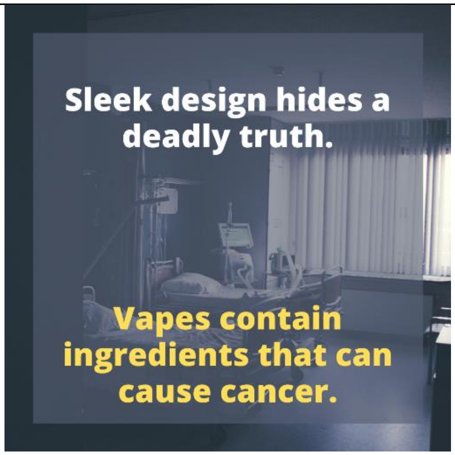	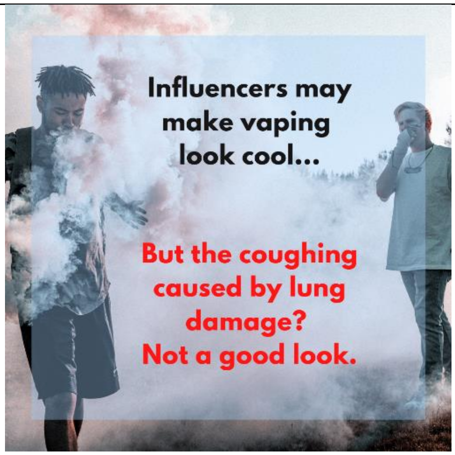	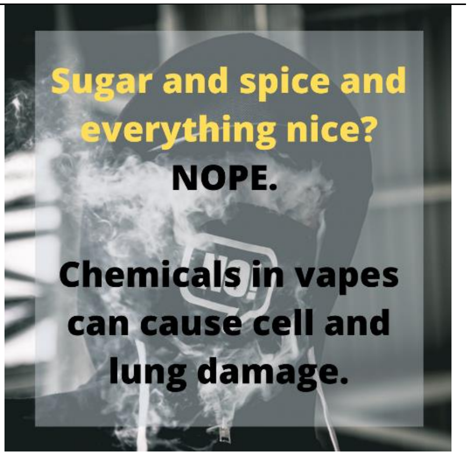
	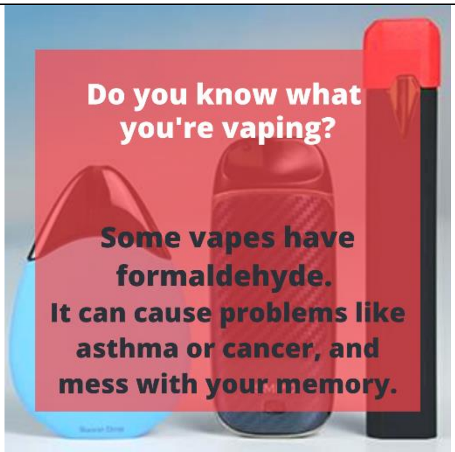	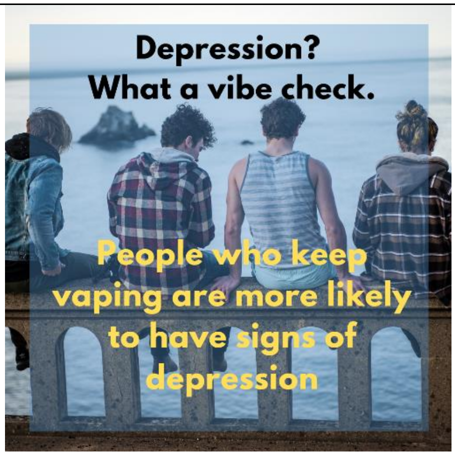	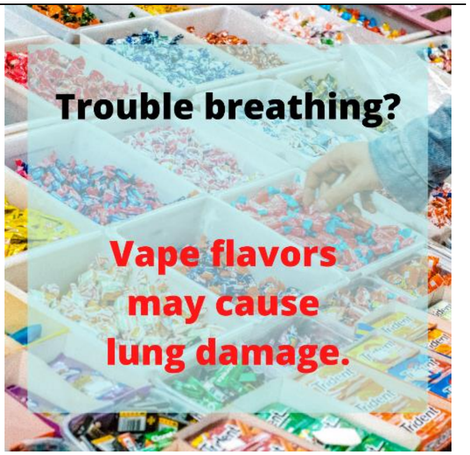

	Alone	+Social	+Flavor
**ADDICTION**		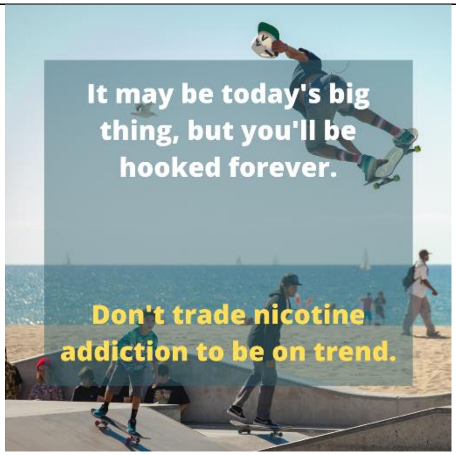	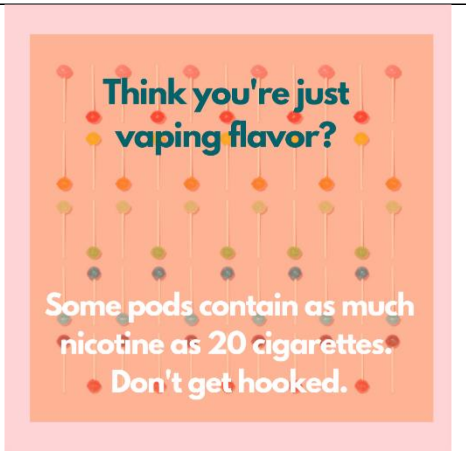

NOTE: Participants in the intervention condition were exposed to all eight messages.

Figure S2. Sun safety messages (Control Condition), PACE Vape Messaging Study, 2020

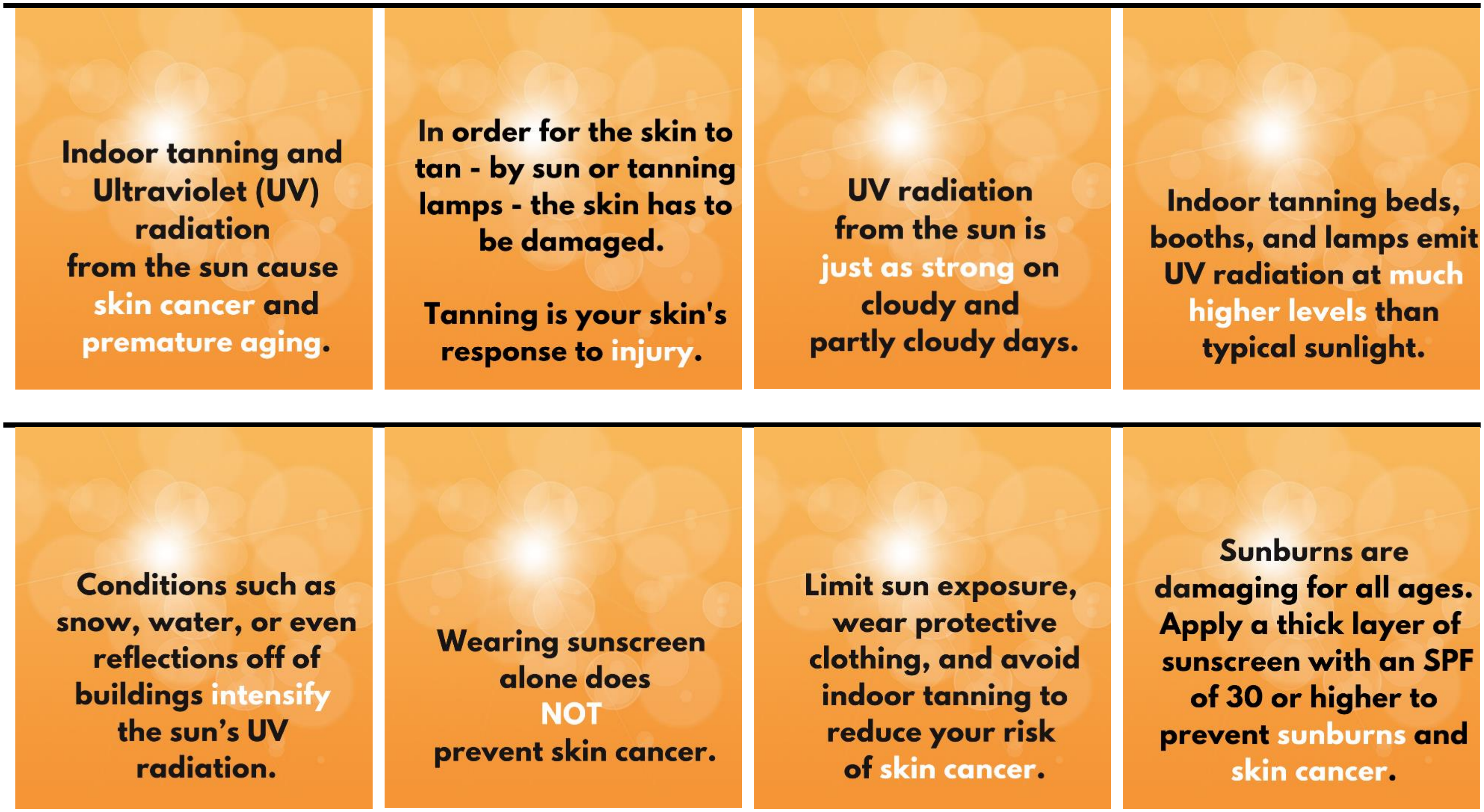

NOTE: Participants in the control condition were exposed to all eight messages.

Table S1. Baseline characteristics of participants retained versus lost to follow-up, PACE Vape Messaging Study, 2020

	**Lost to follow-up n (%)**	**Retained n (%)**	**Total n (%)**	**p-value**
Study condition				0.38
Control	30 (53.6)	242 (47.5)	272 (48.1)	
Intervention	26 (46.4)	268 (52.5)	294 (51.9)	
Sex				0.04
Male	10 (18.2)	160 (31.4)	170 (30.1)	
Female	45 (81.8)	349 (68.6)	394 (69.9)	
Cis or not cisgender				0.12
Cisgender	50 (89.3)	482 (94.5)	532 (94)	
Transgender/don’t know/questioning	6 (10.7)	28 (5.5)	34 (6)	
Sexual identity				0.07
Straight/heterosexual	33 (58.9)	360 (70.6)	393 (69.4)	
Not straight/heterosexual	23 (41.1)	150 (29.4)	173 (30.6)	
Race/ethnicity				0.02
White	51 (91.1)	381 (74.7)	432 (76.3)	
Non-white/other	1 (1.8)	50 (9.8)	51 (9)	
Hispanic	4 (7.1)	79 (15.5)	83 (14.7)	
HRSA-Designated rural county				0.54
No	29 (55.8)	254 (51.3)	283 (51.7)	
Yes	23 (44.2)	241 (48.7)	264 (48.3)	
Employment status				0.65
Work full-time (35 hours/week or more)	18 (32.1)	185 (36.3)	203 (35.9)	
Work part-time (15-34 hours/week)	16 (28.6)	111 (21.8)	127 (22.4)	
Work part-time (<15 hours/week)	10 (17.9)	85 (16.7)	95 (16.8)	
Don't currently work for pay	12 (21.4)	129 (25.3)	141 (24.9)	
Subjective financial status				0.58
Live comfortably	22 (39.3)	172 (33.7)	194 (34.3)	
Meet needs with a little left	23 (41.1)	194 (38)	217 (38.3)	
Just meet basic expenses	10 (17.9)	132 (25.9)	142 (25.1)	
Don't meet basic expenses	1 (1.8)	12 (2.4)	13 (2.3)	
Ever use				

Cigarettes	28 (50)	182 (35.7)	210 (37.1)	0.04
Electronic vapor products (EVP)	38 (67.9)	320 (62.7)	358 (63.3)	0.45
Past 30-day use				
Cigarettes	12 (22.2)	78 (15.3)	90 (16)	0.19
Electronic vapor products (EVP)	18 (32.1)	147 (28.8)	165 (29.2)	0.60
Cigar/cigarillo/little cigar	2 (3.6)	31 (6.1)	33 (5.8)	0.45
Smokeless tobacco	3 (5.4)	5 (1)	8 (1.4)	0.01
Hookah or waterpipe	2 (3.6)	10 (2)	12 (2.1)	0.43
Exposure to tobacco prevention campaigns				
Real Cost	44 (78.6)	331 (64.9)	375 (66.3)	0.07
truth	36 (64.3)	280 (54.9)	316 (55.8)	0.40
Unhyped	11 (19.6)	107 (21)	118 (20.8)	0.85

Table S2. Balance by study condition among those who completed follow-up, PACE Vape Messaging Study, 2020

		Control n (%)	Intervention n (%)	Total n (%)	p-value
Sex					0.86
	Male	77 (31.8)	83 (31.1)	160 (31.4)	
	Female	165 (68.2)	184 (68.9)	349 (68.6)	
Cis or not cisgender					0.20
	Cisgender	232 (95.9)	250 (93.3)	482 (94.5)	
	Transgender/don’t know/questioning	10 (4.1)	18 (6.7)	28 (5.5)	
Sexual identity					0.67
	Straight/heterosexual	173 (71.5)	187 (69.8)	360 (70.6)	
	Not straight/heterosexual	69 (28.5)	81 (30.2)	150 (29.4)	
Race/ethnicity					0.82
	White	178 (73.6)	203 (75.7)	381 (74.7)	
	Non-white/other	24 (9.9)	26 (9.7)	50 (9.8)	
	Hispanic	40 (16.5)	39 (14.6)	79 (15.5)	
HRSA-Designated rural county					0.71
	No	119 (50.4)	135 (52.1)	254 (51.3)	
	Yes	117 (49.6)	124 (47.9)	241 (48.7)	
Employment status					0.17
	Work full-time (35 hours/week or more)	92 (38)	93 (34.7)	185 (36.3)	
	Work part-time (15-34 hours/week)	60 (24.8)	51 (19)	111 (21.8)	
	Work part-time (<15 hours/week)	34 (14)	51 (19)	85 (16.7)	
	Don't currently work for pay	56 (23.1)	73 (27.2)	129 (25.3)	
Subjective financial status					0.27
	Live comfortably	84 (34.7)	88 (32.8)	172 (33.7)	
	Meet needs with a little left	87 (36)	107 (39.9)	194 (38)	
	Just meet basic expenses	68 (28.1)	64 (23.9)	132 (25.9)	
	Don't meet basic expenses	3 (1.2)	9 (3.4)	12 (2.4)	
Ever use					
	Cigarettes	79 (32.6)	103 (38.4)	182 (35.7)	0.17
	Electronic vapor products (EVP)	153 (63.2)	167 (62.3)	320 (62.7)	0.83
Past 30-day use					
	Cigarettes	31 (12.8)	47 (17.6)	78 (15.3)	0.13

Electronic vapor products (EVP)	66 (27.3)	81 (30.2)	147 (28.8)	0.46
Cigar/cigarillo/little cigar	16 (6.6)	15 (5.6)	31 (6.1)	0.63
Smokeless tobacco	5 (2.1)	0 (0)	5 (1)	0.02
Hookah or waterpipe	6 (2.5)	4 (1.5)	10 (2)	0.42
Exposure to tobacco prevention campaigns				
Real Cost	145 (59.9)	186 (69.4)	331 (64.9)	0.08
truth	128 (52.9)	152 (56.7)	280 (54.9)	0.33
Unhyped	46 (19)	61 (22.8)	107 (21)	0.12
